# Patients’ and professionals’ perspectives on the consideration of patients’ convenient therapy periods as part of personalised rehabilitation: a focus group study with patients and therapists from inpatient neurological rehabilitation

**DOI:** 10.1186/s12913-022-07755-3

**Published:** 2022-03-21

**Authors:** Mona Dür, Claudia Wenzel, Patrick Simon, Gerhard Tucek

**Affiliations:** 1 Department of Health Sciences, IMC University of Applied Sciences, Applied Health Sciences Master Degree Programme, Piaristengasse 1, 3500 Krems, Austria; 2 IMC University of Applied Sciences, Josef Ressel Centre for Horizons of personalised music therapy, University of Applied Sciences Krems, Piaristengasse 1, 3500 Krems, Austria; 3 Duervation, Spitalgasse 6/1, 3500 Krems, Austria; 4 Department of Health Sciences, IMC University of Applied Sciences, Music Therapy Bachelor and Master Degree Programme, Piaristengasse 1, 3500 Krems, Austria

**Keywords:** Delivery of health care, Rehabilitation, Quality of health care, Health services research

## Abstract

**Background:**

Research on the optimal period for administering health services, especially rehabilitation interventions, is scarce. The aims of this study were to explore the construct of patients’ convenient therapy periods and to identify indicators based on the perspectives of patients and different health professionals from inpatient neurological rehabilitation clinics.

**Methods:**

This study was part of a larger project on patients’ convenient therapy periods following a mixed methods approach. In the current study a grounded theory approach was employed based on the use of focus group interviews. Focus group interviews were conducted in three different inpatient neurological rehabilitation clinics. Patients and therapists from inpatient neurological rehabilitation clinics who were able to speak and to participate in conversations were included.

**Results:**

A total of 41 persons, including 23 patients and 18 therapists, such as music and occupational therapists, participated in a total of six focus group interviews. The analysis of the focus group interviews resulted in the identification of a total of 1261 codes, which could be summarised in fifteen categories. However, these categories could be divided into five indicators and ten impact factors of convenient therapy periods. Identified indicators were verbal and non-verbal communication, mental functions, physiological needs, recreational needs, and therapy initiation.

**Conclusions:**

The results provide initial evidence that convenient therapy periods are clinically relevant for patients and therapists. Different states of patients’ ability to effectively participate in a rehabilitation intervention exist. A systematic consideration of patients’ convenient therapy periods could contribute to a personalised and more efficient delivery of intervention in neurological rehabilitation. To our knowledge, this study is one of the first attempts to research convenient therapy periods.

## Background

Physical and rehabilitation medicine is a medical speciality that focuses on the improvement of functioning based on a holistic multi-professional teamwork approach in acute, post-acute, post-early and long-term settings [1]. Rehabilitation is a broader concept which refers to “a set of interventions designed to optimize functioning and reduce disability in individuals with health conditions in interaction with their environment” [2].

Due to medical progress, there is an increasing call for personalisation in health care and rehabilitation interventions [3, 4]. In medicine, personalisation commonly refers to medication treatment which is tailored to the individual characteristics of a defined person or group of persons [5]. In rehabilitation personalisation refers to individualized rehabilitation programmes which are tailored to the patients’ health conditions and capabilities [6, 7]. However, literature on personalised, precision or tailored physical and rehabilitation medicine [4, 7] and the different sorts of related therapies such as music therapy [8–10], occupational therapy [11] or physiotherapy [12, 13] is scarce. Literature on personalised or tailored speech therapy does not exist at all. One aspect of personalisation is the appropriate timing of health care and rehabilitation interventions [5].

Besides other factors that determine patient outcomes of rehabilitation interventions, their appropriate timing is crucial, and thus is frequently addressed in health care and research. This is especially true for multi-professional clinical neurologic rehabilitation [14, 15]. For example, the critical window for recovery “a period of heightened plasticity in which the patient seems to be more responsive” to allied health services [16], such as physiotherapy, is essential for the outcome of neurologic rehabilitation services. However, research on the optimal periods for administering multi-professional rehabilitation interventions is scarce. Existing studies have focused on the optimal periods for physical strain in the field of physiotherapy [17], and for sessions in the psychotherapy setting [18] or on the timing and duration of rehabilitation interventions in recovery processes such as in stroke rehabilitation [19–22]. Additionally, attempts have been set to identify right intervals between and intensity of treatment sessions, with the aim to optimize patient outcomes [23]. Other studies focused on weekend allied health services and found positive effects on patient outcomes and costs [22, 24–26]. Furthermore, the temporal structure of the recovery after stroke has been explored [16].

An important construct related to the outcomes of rehabilitation services is patients’ engagement. Patients’ engagement in neurologic rehabilitation was found to improve functional outcomes for clients [27, 28]. Several studies showed that engaged patients achieved significantly better outcomes than nonengaged patients did [27, 29]. Engagement in physical and rehabilitation medicine refers to the patient’s involvement in rehabilitation and healthcare interventions [30]. There might be distinct periods in which patients’ ability to benefit from engagement enhancing interventions varies. However, the literature on engagement does not refer to such periods yet. To summarize, there is some evidence for the existence of distinct periods, where the delivery of treatment is most effective to improve therapy outcome and to reduce long-term impairment in neurologic rehabilitation.

There is no concept that describes a distinct period in which rehabilitation interventions within neurologic rehabilitation would have their greatest effects based on patients’ momentary ability to engage. However, the authors assume that these distinct and convenient periods for rehabilitation interventions do exist [31] that are called convenient therapy periods within this article. Based on their practical experience, the authors also assume that the ability to optimally benefit from a rehabilitation intervention might depend on patients’ time-limited enhanced responsiveness to the interventions and varies during a day.

Knowledge about patients’ convenient therapy periods and their indicators could help clinicians to identify and consider these periods in their clinical practice. The consideration of patients’ ability to respond or engage in rehabilitation interventions might have a positive effect on patients’ ability to benefit from rehabilitation interventions. Moreover, the consideration of patients’ convenient therapy periods could improve the effects of rehabilitation interventions, improve patient outcomes, and thereby save costs [22, 32].

Furthermore, it might be important to relate patients and health professionals’ preferences and perspectives to structural and organizational conditions of therapy [33, 34]. In clinical practice a consideration of convenient therapy periods in the scheduling and timing of therapy sessions could contribute to more suitable and effective music therapy, occupational therapy, speech therapy or physiotherapy [23, 35].

The aims of this study were to explore the construct of patients’ convenient therapy periods and to identify indicators based on the perspectives of patients and different health professionals from inpatient neurological rehabilitation clinics.

## Methods

This study was part of a larger project on patients’ convenient therapy periods following a mixed methods approach [31, 36, 37]. In the current study a grounded theory approach was employed based on the use of focus group interviews [38, 39], due to the absence of existing literature on the construction and definition of patients’ convenient periods for rehabilitation interventions. Grounded theory as a research approach includes iterative analyses, going back and forth the data, and encompasses comparison of the analysis and the original data [38, 39].

### Participants

Patients and therapists from three inpatient neurological rehabilitation clinics were recruited for this study by “Theoretical Sampling”, a specific grounded theory sampling approach, seeking pertinent data to develop the emerging theory [38]. Sample size was based on theoretical sampling. To fulfil the criteria for inclusion patients had an age of ≥18 years, were in phase c (post-early) rehabilitation, defined as phase in which patients are “cooperative but dependent for selfcare” [40, 41], and had already experienced two or more different rehabilitation interventions (e.g., occupational therapy and music therapy) at the time point of data collection. Additionally, patients had sufficient language skills, as well as mental and physical abilities and willingness to participate in a focus group. Therapists were included if they had worked at an inpatient neurological rehabilitation clinic for at least 1 year, had sufficient language skills and were willing to participate. Patients of different sex, age, and health conditions and/or diagnoses (e.g., stroke) and a wide range of therapists, including art therapists, music therapists, occupational therapists, physiotherapists, and speech therapists, were asked to participate.

### Data collection

Participants received both verbal and written information on the study from the local study coordinators or principal investigators at the institution (names are not shown to ensure participants anonymity and confidentiality). Sex and age of all participants were recorded, as well as diagnosis and disease duration in the case of patients, and years of work experience and profession in the case of therapists. Focus group interviews were used to identify determinants of convenient therapy periods based on the perspectives of patients and therapists from inpatient neurological rehabilitation clinics. Focus group interviews are systematic discussions between individuals experiencing a specific phenomenon to gather insights into their experiences and perspectives concerning the issue of interest [42]. Focus group interviews are frequently used to explore patients’ and health professionals’ perspectives in rehabilitation research [34, 43]. Focus group interviews are led by a moderator who asks questions related to the specific focus. The content of the focus group interviews can be diverse and may run contrary to the expectations and presumptions of the focus group moderator and/or researcher. Compared to focus group interviews [44], one-to-one interviews can be restricted to the content directly asked by the interviewer and/or raised by the interviewee.

Local study coordinators organised dates, timeframes and conference rooms for the focus group interviews. In each inpatient neurological rehabilitation clinic, two focus group interviews, one with patients and one with therapists were conducted in spring 2017. The focus group interviews were led by one researcher, experienced in conducting focus group interviews (MD [PhD] or CW [Dr. phil.]) and assisted by another researcher (PÖ [Mag.], IZ [MSc], MD [PhD] or CW [Dr. Phil.]). One of these researchers (CW) knew one of the participating therapists prior to study commencement. The focus group interviews were audio recorded and transcribed verbatim. Two audio recorders were placed on a table in the centre of the group. People who were not as involved in the conversation during the focus group were explicitly asked and invited to share their perspectives of the moderator.

### Data analysis

Based on the grounded theory approach we used the so called constant comparative method and went through an iterative analysis process [38]. Firstly, the main analysts (MD and CW) delved into the interview transcripts to get an overview of the collected data. Secondly, initial codes were created from the data by extracting the content of every single proposition of the participants. Initial codes were single or several words, which referred to the content and the meaning of text sequences of the interview transcripts. Thirdly, focused coding and categorizing was employed in joint sessions by the two analysts (MD and CW). Most significant and frequent initial codes were sorted and synthesized into tentative categories, aggregates of interrelated codes. A constant comparison of categories, codes and original data allowed an evaluation of the relative usefulness of the empirically grounded core conceptual categories and an identification and exploration of their interrelations (MD and CW). Fourthly, based upon original quotes indicators for convenient therapy periods were identified and written down, discussed, and reflected in an interdisciplinary team of health professionals and researchers to enhance trustworthiness and credibility of data analysis. The team consisted of different health professionals and researchers from anthropology, general practice, linguistic science, music therapy, occupational therapy, and psychology, skilled and experienced in the use of qualitative research methods.

### Ethical considerations

All participants received information about the study and gave written and oral informed consent for participation. The study was approved by two local ethics commissions, responsible for the two different states for the inpatient clinics in Austria. The study complies with the Declaration of Helsinki. In the given examples, pseudonyms were used to guarantee anonymity of the participants. Detailed and centre specific information about the focus groups content was not conveyed to the clinic staff members. The funders played no role in the design, conduct, or reporting of this study.

## Results

### Participants

A total of 41 persons, including 23 patients and 18 therapists, participated in a total of six focus group interviews. Demographic data of the participants are presented in Table 1.

**Table 1:** Demographic data

Patients		Therapists	
Total	23	Total	18
Female *n* (%)	7 (30)	Female *n* (%)	14 (78)
Male *n* (%)	16 (70)	Male *n* (%)	4 (22)
Median age in years (IQR)	54 (48–75)	Median age in years (IQR)	37 (28–43)
Health condition		Profession	
Epilepsy *n* (%)	2 (9)	Music therapist *n* (%)	2 (11)
Multiple sclerosis *n* (%)	3 (13)	Occupational therapist *n* (%)	5 (28)
Vascular syndromes of brain in cerebrovascular diseases including stroke *n* (%)	10 (44)	Physiotherapist *n* (%)	6 (33)
Cervical disc disorders *n* (%)	1 (4)	Speech therapist *n* (%)	4 (22)
Unspecified rheumatism *n* (%)	1 (4)	Art therapist *n* (%)	1 (6)
Difficulty in walking *n* (%)	4 (18)	Median work experience in years (IQR)	5 (2–11)
Intracranial injury *n* (%)	1 (4)		
Sensorimotor impairment *n* (%)	1 (4)		
Median time since onset in years (IQR)	1 (0–5)		

*IQR* interquartile range, *n* number, *%* percentage

**Fig. 1:**
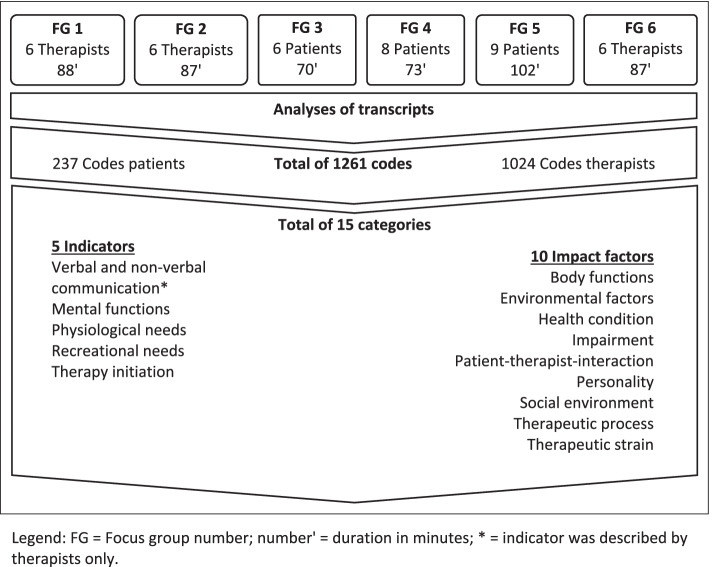
Overview of focus groups and results*.*

The six focus group interviews had a mean duration of 85 min and six to nine participants. Further details on the focus group interviews are presented in Fig. 1.

### Categories and Indicators

The analysis of the focus group interviews resulted in the identification of a total of 237 codes for the patient focus groups and 1024 codes for therapist focus groups, which could be summarised in fifteen categories. However, these categories could be assigned to five indicators and ten impact factors of convenient therapy periods as presented in Fig. 1. Indicators were categories that have been described to imply patients’ momentary ability to benefit from a therapy session. Impact factors were categories that have been described to affect patients’ momentary ability to benefit from a therapy session. According to the study aim, we present those categories in the following, which were identified as indicators. Subsequently, each indicator is defined and substantiated by original quotes, as presented in Tables 2, 3, 4, 5, 6.

**Table 2:** Original quotes as example for the indicator verbal and non-verbal communication

For example, *verbal communication* was identified when Tamara talked about her experiences of patients introducing themselves to her. “(…) w*hen a patient starts the first session saying: ‘I was trained in special exercises at the clinic. I practice them daily.*’ *Or they say*: ‘*I’ve practised them intensely*’. *Then I’m sure the patient is ready to participate in my therapy session*” (Tamara, 28, speech therapist, 7 y, 1: 119). Franziska reported on patient feedback after the session: “[The patients] *just express how they felt about a* [therapy] session, for instance, it was fun, I really enjoyed it or it was absolutely fantastic” (Franziska, 32, physiotherapist, 3 y, 6: 134). Non-verbal communication was identified when a physiotherapist reflected upon various patient signals: “*You can quite clearly see how a patient is responding to a particular exercise* [during a physiotherapy session], *so you check the pulse and oxygen saturation*. *And of course, you notice the patient’s complexion and facial expression*” (Nina, 42, physiotherapist, 22 y, 1: 507). However, verbal, and non-verbal communication was described as indicator for convenient therapy periods from therapists only.	

*italic* = original illustrative quote, (therapist’s pseudonym, numbers = age in years, profession, number y = years of practice; focus group number: number of paragraphs within Atlas. Ti, Vers. 7.0)

**Table 3:** Original quotes as example for the indicator mental functions

A physiotherapist referred especially to the aspects of the limited drive, attention, and vigilance in the patients: “*In fact, many of our patients have limited drive, attention and vigilance*” (Cornelia, 53, physiotherapist, 12 y, 1: 157). However, for a music therapist the patient’s level of attention is crucial for therapy: “*I realize their level of attention when they come to me, whether or not they are ready* [for therapy]” (Melitta, 28, music therapist, 1 y, 1: 131). Patients referred to the importance of mental functions in the following way. Martin, a stroke patient, related his own experience of motivation and drive function in his own words: “*I managed to get back my motivation gradually. I had just been through a low (point) and for two or three days I didn’t even want to see a therapist*” (Martin, 56, patient, 1 y, 5: 242). A patient with rheumatism points out to the relevance of time of day concerning his readiness for any kind of therapy: „*I feel a lot more motivation in the afternoon, as that’s when I’m in the right* [physical] *state* [for therapy]” (Gernot, 19, patient, < 1 y, 5: 174). However, another patient with sensorimotor impairment emphasises the importance of self-motivation for therapy sessions at any given time: „*I think you have to get yourself in the right mood, don’t you? When you look at the schedule, you see ‘So I’ve got such a session at such a time’, then you’ve just got to get yourself in the right mood*” (Thomas, 50, patient, 2 y, 3: 272).	

*italic* = original illustrative quote, (participant’s / therapist’s pseudonym, numbers = age in years, patient/profession, number y = years since onset/years of practice; focus group number: number of paragraphs within Atlas. Ti, Vers. 7.0)

**Table 4:** Original quotes as example for the indicator physiological needs

For example, Irma, an occupational therapist agreed with a speech therapist and a physiotherapist upon the importance of meeting physiological needs: “(…) *basic needs of patients such as hunger or sleep have to be satisfied*; *otherwise, therapy won’t work*” (Irma, 37, occupational therapist, 15 y, 6: 636). “*And Restroom*” (Tatjana, 54, speech therapist, 30 y & Franziska, 32, physiotherapist, 3 y, 6: 638–642). Saki, a music therapist, referred to patients who are assigned to her close to lunch time: “[they] *are already thinking about lunch and are afraid that they will not get any lunch, if they are late. And then they want to leave therapy earlier and I can’t do anything about that*” (Saki, 41, music therapist, 1.5y, 2: 64). Daniel, a physiotherapist, describes a similar situation with lunchtime: “*When you want to start your therapy exactly when lunch is coming, ((laughs))* […] *and the patient says, ‘my food has just arrived’, then the patient will not join me for therapy*” (Daniel, 33, physiotherapist, 10 y, 6: 436). Irma emphasized that it is also important for patients to have a small break after they had breakfast or lunch, so that the patient “*does not have food in his/her mouth, when the therapist enters the room and wants to start*” (Irma, 37, occupational therapist, 15 y, 6: 302).	

*italic* = original illustrative quote, (participant’s / therapist’s pseudonym, numbers = age in years, patient/profession, number y = years since onset/years of practice; focus group number: number of paragraphs within Atlas. Ti, Vers. 7.0)

**Table 5:** Original quotes as example for the indicator recreational needs

Recreational needs were identified when Nina, a physiotherapist, described the following situation: “*Not every patient says, ‘I am tired’ or ‘I can’t go on any longer’. Yet we do sometimes even hear such statements.* (…) *When a patient climbs on the exercise bike we very frequently hear ‘I have already done so much today, who knows if I’ll still be able to get anything else done.’”* (Nina, 43, physiotherapist, 22 y, 1: 365). Another example is the experience from an occupational therapist called Irma: “*There was a patient who had suffered a stroke and slept all day long. I knew he was willing to participate* [in the therapy]*, but he was so tired. The more demanding it* [the therapy] *was, the more tired he got. You just have to accept that he needs to get some rest. You can get him involved as soon as he is ready*” (Irma, 37, occupational therapist, 15 y, 6: 212–214). This quotation is consistent with the following statement from Thomas, a patient with sensorimotor impairment: “*And then you’re not productive during the session and you get such situations when you think ‘It’s really just not the right time. I could have done a relaxation session instead’*” (Thomas, 50, patient, 2 y, 3: 404).	

*italic* = original illustrative quote, (participant’s / therapist’s pseudonym, numbers = age in years, patient/profession, number y = years since onset/years of practice; focus group number: number of paragraphs within Atlas. Ti, Vers. 7.0)

**Table 6:** Original quotes as example for the indicator therapy initiation

For example, Theodor reported the importance of initial greeting: “*A lot depends just on the way they say hello and the way they say goodbye. It is almost the same as with a job interview. It’s a bit of a funny comparison, but* … (laugh)” (Theodor, 48, occupational therapist, 2 y, 2: 216). Another occupational therapist referred to the importance of therapy initiation in the following way: “*For instance; if* [a patient] *pulls a face when I arrive or if as soon as I reach her bedside, she suddenly seems to be asleep, then everything is clear*. *Then I understand that she is not in the* [right] *mood*” (Heidi, 23, occupational therapist, 1 y, 6: 348). Patients referred to therapy initiation in different ways. Some patients were looking forward to participating in therapy sessions, like Irene, a stroke patient who said: „*I’d always look forward to the physiotherapy sessions, as I’d expected to learn something new again every time*” (Irene, 75, patient, < 1 y, 4: 322). Whereas other patients stressed the fact, that on some days they are not in the mood for any kind of therapy schedule such as a patient with multiple sclerosis: “*Some days I somehow just don’t feel like going to therapy. You know, you get up in the morning and you think ‘Oh God, I’m really not looking forward to that I have to do this and then I have to do that*” (Maria, 46, patient, 9 y, 3: 184).	

*italic* = original illustrative quote, (participant’s / therapist’s pseudonym, numbers = age in years, patient/profession, number y = years since onset/years of practice; focus group number: number of paragraphs within Atlas. Ti, Vers. 7.0)

### Verbal and non-verbal communication

In the current study, communication included verbal and non-verbal communication. Verbal and non-verbal communication was described as indicator for convenient therapy periods from therapists only. Patients and therapists frequently talked about fatigue, exhaustion, and pain, as well as therapy related attitudes, enthusiasm, interest, motivation, and readiness. Therapists focused on non-verbal signals, such as complexion and facial expressions, gestures, muscle tone, posture, and transpiration. Table 2 contains selected original quotes as examples for verbal and non-verbal communication from patients and therapists.

Therapists emphasised the importance of one specific aspect of communication: They reported to be particularly attentive, both at the beginning and during a therapy session, to patients’ signals of their ability to benefit from the session. The information gathered at the very beginning of a specific therapy session is used to determine the characteristics of the rehabilitation intervention. The information gathered during the session allows therapists to adjust these characteristics to the changing ability of the patients to engage. Therapists reported that patients present different manifestations of their ability to benefit from the session and that this ability can be influenced. Some of these manifestations are amenable to improvement by rehabilitation intervention; others are not and are unlikely to change during the session.

### Mental functions

Mental functions included commitment, arousal, attention, consciousness, emotions, energy and drive functions, impulse, motivation, and vigilance. Table 3 contains selected original quotes from patients and therapists as examples for mental functions.

### Physiological needs

Physiological needs included basic needs of patients like hunger, thirst, and toileting needs, which can impede therapies when they are unfulfilled. Table 4 contains selected original quotes on physiological needs from therapists only, because patients did not address this topic directly in the focus group interviews.

### Recreational needs

Recreational needs included needs for pauses, recreation, relaxation, rest, and sleep. Table 5 contains selected original quotes from patients and therapists as examples for recreational needs.

### Therapy initiation

Therapists highlighted the importance of the very first moments of a therapy session and mentioned giving special attention to mood and body language. Therapists reported that patients responded either with a display of interest or lack of interest at the very beginning of a therapy session. Table 6 contains selected original quotes from patients and therapists as examples for therapy initiation.

## Discussion

In the current study, we identified five indicators of convenient therapy periods based on the perspectives of patients and health professionals in neurorehabilitation. Other studies highlighted the importance of knowledge and consideration of the right period to provide specific health services [16–21, 33, 45–48]. The identified indicators have already been explored in health care research, but – to our knowledge – not in relation to convenient therapy periods.

The meaning of communication in the therapeutic setting is well researched. The content of communication relevant to this study included expressions of different aspects such as fatigue, pain, interest, or motivation. Of course, these aspects have been targets of numerous health care interventions [49]. However, their meaning for the therapeutic progress has not been researched so far.

Considering patients’ verbal and non-verbal signals, therapists highlighted the importance of the very first moments of a therapy session as part of rehabilitation interventions, where patients were found to respond either with a display of interest or lack of interest. This attention of therapists to patients’ signals at the very beginning of a therapy session may be related to the phenomenon of attunement. Attunement refers to a process encompassing therapists’ ability to perceive and to respond to patients’ inner state [50–53]. Attunement was found to be relevant in different sorts of rehabilitation interventions, such as music [54, 55] and occupational therapy [56]. However, attunement and convenient therapy periods have not been related so far.

The evaluation of mental functions is routine within therapeutic practice. For example, mental functions are commonly assessed to identify need for treatment or to evaluate the outcome of health care and rehabilitation interventions [57–59]. However, mental functions are assessed to determine impairment and therapy outcomes [59, 60], but not as indicators for patients’ momentary ability to benefit from a therapy session.

The detection and consideration of patients’ physiological needs is important in health care, especially when working with neurorehabilitation patients, who may not be able to express these needs clearly. For example, nurses and therapists, like occupational therapists, generally consider patients’ thirst, hunger, urge to use the toilet and any other physiological needs that may arise [61, 62]. However, the inclusion of physiological needs as indicators for convenient therapy periods has not been reported yet.

Recreational needs have been connected to spinal cord injury patients’ attendance of scheduled therapy sessions [63]. Fatigue was found to be one of the most common reasons for leaving out therapy sessions during inpatient rehabilitation. Another reason given was lack of patient readiness including “being unavailable” or “refusing recreational therapy sessions”. Patients left out an average of 20 h of their therapy during their inpatient rehabilitation [63]. Leaving out therapy sessions could be related to patients’ convenient therapy periods and therapy progress.

There seem to be (adaptive) states in which therapists were able to facilitate patients’ ability to benefit from the therapy session, by adjusting the therapeutic strain through using activating, motivating, or relaxing techniques. However, therapists also reported about patients who seemed to be in (stable) states of inconvenient therapy periods in which the delivery of rehabilitation interventions had not the desired effect. Patients’ adaptive states were reported previously in terms of their engagement in rehabilitation interventions. There is evidence, that patients’ engagement could be enhanced by therapists during rehabilitation. Strategies which were found to enhance patients’ engagement included interventions that promote trust, rapport, empowerment, and motivation [27, 64]. Patients’ ability to benefit from the therapy session might also be influenced by the therapeutic relationship [65] and patients’ engagement, which however needs further research.

Within the current study, we obtained initial knowledge that might influence therapeutic clinical practice in neurologic rehabilitation [66] and contribute to an increased consideration of convenient therapy periods in terms of flexible scheduling and conduction of health care services. This might have a positive impact on the outcomes of neurological rehabilitation services [33, 66, 67], patients’ satisfaction, the number of missed therapies and costs [63].

A systematic evaluation of patients’ convenient therapy periods could enable therapists to deliver a more personalised and efficient delivery of neurological rehabilitation services [68]. Consequently, a measurement instrument is needed to assess and address convenient therapy periods in the clinical practice. This measurement instrument is being developed and researched as part of a larger research project on patients’ convenient periods for rehabilitation interventions [31, 36, 37]. However, the follow-up studies are not part of this paper and will be published in the future. Therefore, an increased consideration of convenient therapy periods in the scheduling and conduction of rehabilitation interventions as well as a systematic assessment of patients’ convenient therapy periods in clinical practice is recommended.

### Limitations

This study has several strengths and limitations. Data was purposeful and included three different rural inpatient neurological rehabilitation clinics, located in two federate states of Austria. The inclusion of additional and urban inpatient neurological rehabilitation clinics could have led to other findings. Furthermore, the current study focused on convenient therapy periods of patients from neurological rehabilitation. Indicators for convenient therapy periods could differ between patients with diverse health conditions. Additionally, participants included patients with sufficient concentration and communication skills, and those who were transferable to the rooms of the focus groups, exclusively. Consequently, further research is needed to explore indicators for convenient therapy periods from the perspectives of bedridden patients and patients with limited concentration and communication skills. This study was part of a larger project on patients’ convenient therapy periods following mixed methods approach. However, mixed-methods studies often lack a detailed description of used methods [69]. Therefore, preliminary results of the analysis of only one part of the data collection and analysis are presented within this paper.

## Conclusions

The findings of the current study provide first insights into convenient therapy periods and encourage the initiation of a scientific discourse on convenient therapy periods and their increasing consideration in neurological health service and research. A systematic consideration of patients’ convenient therapy periods could contribute to a personalised and more efficient delivery of intervention in neurological rehabilitation.

## Data Availability

The datasets generated and analysed during the current study are not publicly available due to data protection law but are available from the corresponding author on reasonable request.
